# Management of Ductal Carcinoma In Situ: An Ontario Health (Cancer Care Ontario) Clinical Practice Guideline

**DOI:** 10.3390/curroncol31120569

**Published:** 2024-12-03

**Authors:** Muriel Brackstone, Lisa Durocher-Allen, Nadia Califaretti, Andrea Eisen, Sarah Knowles, Abeer Salim, Taude Plexman, C. Anne Koch

**Affiliations:** 1 London Health Sciences Centre-London Regional Cancer Program, Victoria Hospital, 800 Commissioners Road East, London, ON N6A 5W9, Canada; 2 Program in Evidence-Based Care, Ontario Health (Cancer Care Ontario), 699 Concession Street, Hamilton, ON L8V 1C3, Canada; 3 Grand River Regional Cancer Centre, 835 King Street W, Kitchener, ON N2G 1G3, Canada; nadia.califaretti@grhosp.on.ca; 4 Sunnybrook Odette Cancer Centre-Medical Oncology, T2-038-2075 Bayview Ave, Toronto, ON M4N 3M5, Canada; andrea.eisen@sunnybrook.ca; 5 Department of Surgery, St Joseph’s Health Care Centre, 268 Grosvenor Street, London, ON N6A 4V2, Canada; sarah.knowles@lhsc.on.ca; 6 Patient and Family Advisor, Ontario Health, 500-525 University Ave, Toronto, ON M5G 2L3, Canada; 7 Princess Margaret Hospital, 610 University Ave, Toronto, ON M5G2M9, Canada; anne.koch@uhn.ca

**Keywords:** guideline, surgery, radiation therapy, endocrine therapy, Ductal Carcinoma in Situ

## Abstract

(1) Background: To make recommendations on the most effective therapy options for Ductal Carcinoma of the Breast (DCIS) patients; (2) Methods: MEDLINE, EMBASE, Cochrane Library, PROSPERO databases, and main relevant guideline websites were searched. Draft versions of the guideline went through formal internal and external reviews, with a final approval by the Program in Evidence Based Care and the DCIS Expert Panel. The Grading of Recommendations, Assessment, Development, and Evaluation approach was followed; (3) Results: Based on the current evidence from the systematic review and this guideline authors’ clinical opinions, initial draft recommendations were developed to improve the management of patients with DCIS. After a comprehensive internal and external review process, ten recommendations and 27 qualifying statements were eventually made. This guideline includes recommendations for the primary treatment of DCIS with surgical treatment and/or radiation therapy and the management of DCIS after primary treatment for patients with DCIS, including DCIS with microinvasion (<1 mm through the duct); (4) Conclusions: The current guideline was created after a systematic review and a comprehensive internal and external review process. We believe this guideline provides valuable insights that will be useful in clinical decision making for health providers.

## 1. Introduction

Ductal Carcinoma in Situ (DCIS) is a noninvasive breast cancer where the neoplastic cells are contained within the milk ducts of the breast. Many DCIS lesions are nonpalpable and are often identified at the time of routine screening mammography. There has been an increase in the diagnoses of DCIS concurrent with the use of routine screening mammography. Among new diagnoses of breast cancer detected through screening, DCIS accounted for approximately one-fifth [1,2].

In 2018, the Breast Disease Site Group in Ontario collaborated with the Program in Evidence-Based Care (PEBC) of Ontario Health (Cancer Care Ontario) to develop a clinical practice guideline (CPG) on DCIS [3]. Since its development, there have been advances in the treatment options available to DCIS patients. There is new evidence, for example, on the option of hypofractionation and/or shorter duration of radiation (RT), hormonal therapy options, and the emerging area of molecular profiling. As such, the Working Group Clinical Practice Guideline (CPG) authors (with expertise in medical oncology, radiation oncology, surgical oncology, and health research methodology), along with patient representatives and in association with the PEBC of Ontario Health (Cancer Care Ontario) and the DCIS Guideline Development Group updated and broadened the scope of the 2018 CPG. The objective is to systematically review the medical literature and develop recommendations on the most effective therapy options for DCIS patients.

## 2. Materials and Methods

### 2.1. Background

This evidence-based and evidence-informed guideline used the methods of the Practice Guideline Development Cycle [4,5]. This process includes conducting a systematic review, the Working Group interpreting the evidence, and drafting recommendations, followed by an internal review process by content and methodology experts, Ontario clinicians, and other stakeholders completing the external review process. Further details on the methods and the systematic review are described on the Ontario Health (Cancer Care Ontario) website [6].

### 2.2. Research Questions

#### 2.2.1. Primary Treatment of DCIS: Surgical Treatment

• What is the optimal surgical treatment (breast conserving surgery [BCS]; mastectomy; active surveillance) for patients with DCIS when considering disease-free survival (DFS), recurrence, and significant complications after surgery (i.e., bleeding or infection)?
• What margin width minimizes the risk of recurrence and complications after surgery (i.e., bleeding, infection) and increases DFS in patients undergoing DCIS receiving BCS or mastectomy?
• After the initial surgery of BCS or mastectomy with suboptimal margin width (close or positive), should re-excision be considered to improve DFS, recurrence, and reduce complications after surgery, defined as a complication requiring reoperation within 30 days (i.e., bleeding or infection)?

#### 2.2.2. Primary Treatment of DCIS: Surgical Treatment and/or RT

• Should molecular profile testing be added to clinical evaluation to guide the use of adjuvant therapy in patients with DCIS?
• In DCIS patients who have undergone BCS or mastectomy, should breast irradiation be offered to improve DFS and reduce recurrence with acceptable adverse events of irradiation?

#### 2.2.3. Management of DCIS After Primary Treatment

• In DCIS patients who have undergone BCS or mastectomy, what is the role of endocrine therapy in the management of DCIS to improve DFS and reduce recurrence (invasive or noninvasive) and contralateral events with acceptable treatment adverse events?

### 2.3. Target Population

These recommendations apply to women with DCIS, including women with DCIS with microinvasion (DCIS-M) (<1 mm through the duct).

### 2.4. Intended Users

Intended users of this guideline are clinicians and other healthcare professionals involved in the management of patients with DCIS.

### 2.5. Literature Search

MEDLINE, EMBASE, PROSPERO, and the Cochrane Library were screened for existing systematic reviews, original studies, abstracts, and systematic review-based guidelines that were relevant to the research questions from January 2018 to 27 November 2023. In addition, the main relevant guideline websites (National Institute for Health and Care Excellence Evidence Search, Scottish Intercollegiate Guidelines Network, American Society of Clinical Oncology, National Health and Medical Research Council–Australia Clinical Practice Guidelines Portal, Cancer Council Australia, Geneva Foundation for Medical Education, and Research, American Society for Radiation Oncology (ASTRO), American College of Radiology, and Alberta Health Services) were searched from 1 January 2019 to 1 August 2022. On 26 July 2023, the National Institute Clinical Trials Database was also searched for ongoing, unpublished, or incomplete studies. Relevant existing guidelines were assessed using the Appraisal of Guidelines for Research and Evaluation (AGREE II) tool [7], and only those that scored 50% in the rigor of development domain, which evaluates the guideline’s methodological quality, were included.

Of the 3404 studies found in the literature search, five systematic reviews [8,9,10,11,12] and 22 primary studies [13,14,15,16,17,18,19,20,21,22,23,24,25,26,27,28,29,30,31,32,33,34] met the predefined eligibility criteria (Appendix A.1). The risk of bias of relevant systematic reviews was assessed with the ROBIS tool [35], and only those with a low risk of bias ratings were included. As a result, two of the five systematic reviews were excluded due to the risk of bias concerns [8,11]. The risk of bias for randomized studies was assessed using the Cochrane risk of bias (RoB2) tool for randomized controlled trials (RCTs) [36] and ROBINS-I for non-RCTs [37]. The certainty of the evidence per outcome for each research question was assessed using the Grading of Recommendations, Assessment, Development, and Evaluation (GRADE) approach. The aggregate certainty of each comparison of interventions ranged from very low to high after considering the risk of bias, inconsistency, indirectness, imprecision, and publication bias. Further details on the GRADE evaluations, risk of bias assessments, and summary of findings tables are described here [6].

### 2.6. Recommendation Development and Review

The Working Group evaluated the results from the systematic review and developed clinical practice recommendations. The document was then reviewed by the Expert Panel (five medical oncologists, two surgical oncologists, four radiation oncologists, and two pathologists in Ontario) and the PEBC Report Approval Panel, a three-person methodology expertise panel. Conflict of interest forms were signed by all internal reviewers (Appendix A.2). All participants approved the document, and their comments were discussed by the Working Group in revising the document.

Targeted Peer Reviewers from Ontario who are considered clinical and/or methodological experts were identified during the guideline development process and agreed to participate in the review. Conflict of interest forms were signed by all Targeted Peer Reviewers (Appendix A.2). Responses were received on 31 January 2024, and key results from the questionnaire are summarized in Appendix A.3. Through Professional Consultation, all relevant healthcare professionals and stakeholders from the PEBC database (across Canada, but primarily Ontario) who are the intended users of the guideline were sent a brief online survey. Thirty-two provided feedback on the draft guideline recommendations, and their responses are summarized in Appendix A.4. The Working Group considered all feedback when finalizing the guideline.

## 3. Recommendations and Key Evidence

### 3.1. Primary Treatment of DCIS: Surgical Treatment

#### 3.1.1. Recommendation 1

• Women with DCIS of the breast (with or without microinvasion) who are candidates for BCS should be offered the choice of BCS or mastectomy with the option of reconstruction. The decision of whether to have one surgery over another should be made in consultation with the patient and should consider the balance of benefits and risks and patient preferences.

#### 3.1.2. Qualifying Statements for Recommendation 1

• Benefits and harms may vary depending on patient and disease characteristics such as patient factors/comorbidities, patient preferences, tumor characteristics, life expectancy, and any contraindication to or unwillingness to receive RT.
• When BCS is performed, all mammographically suspicious calcifications should be removed, and the margins should be microscopically cleared of DCIS. RT options after BCS are described in Recommendation 5 below.
• The option of immediate lumpectomy reconstruction in the case of BCS should be offered if a patient is deemed an appropriate candidate.
• Patients eligible for genetic testing should be referred so that results may be considered before a surgical treatment plan is finalized (this may include a bilateral risk-reducing mastectomy).
• Active surveillance is an area of ongoing investigation; it is not a standard option currently. This might be an area of consideration for certain patients.
• The use of imaging modalities to assess for residual disease in patients with positive markings post BCS is outside the scope of this guideline. The Working Group consensus favors positive margins being treated surgically, given the perceived low sensitivity for detecting residual disease versus postoperative changes in patients having undergone recent surgery with all imaging modalities.

#### 3.1.3. Key Evidence and Justification for Recommendation 1

There were no randomized controlled trials (RCTs) that met the inclusion criteria comparing BCS versus mastectomy; therefore, no strong evidence for one treatment strategy over another is currently available. This recommendation, with its Qualifying Statements, was made through the consensus of the Working Group that patients and their healthcare provider team should discuss management strategies, and the patient should be offered the choice of BCS or total mastectomy with the option of reconstruction. This is consistent with the recommendations from another consensus guideline [38]. Patients are eligible for BCS when, after removing disease tissue, there remains enough tissue to leave the patients with a cosmetically acceptable breast mound. The option of immediate breast conserving oncoplastic remolding or reconstruction should be offered if a patient is deemed an appropriate candidate. Recognizing that BCS can be offered in conjunction with RT, patients choosing not to receive RT might select mastectomy as their surgical treatment. These recommendations place a high value on patients’ individual surgical preferences after reviewing the benefits and risks of either BCS and total mastectomy with or without immediate or delayed reconstruction. Active surveillance as an alternative to surgical treatment in DCIS patients has several ongoing RCTs comparing active surveillance and conventional surgical treatment, such as the LORIS trial (UK-LORIS), the LORD trial (NCT02492607), and the COMET trial (NCT02926911; see Appendix A.5).

#### 3.1.4. Recommendation 2

• In patients undergoing BCS or mastectomy, a margin width of at least 2 mm is optimal to minimize the risk of local recurrence (LR).

#### 3.1.5. Qualifying Statements for Recommendation 2

• It remains entirely appropriate in pathology practice to report only DCIS at the inked margin as “positive” and to provide distance to the closest margin(s) when margins are negative.
• DCIS-M should be considered as DCIS when considering the optimal margin width and additional surgery.
• Patients who have close or positive margins are directed to the recommendations on the benefits of re-excision prior to receiving RT.

#### 3.1.6. Key Evidence and Justification for Recommendation 2

Two systematic reviews [9,10] were found, and the risk of bias in those publications was deemed low using the ROBIS assessment tool. In addition, three comparative studies (with or without RT) were identified [23,29,31]. The level of certainty regarding the evidence for each comparison was classified as “very low”. The Working Group, including two patient representatives, were unanimous in their opinion that patients would value a decreased risk of recurrence and an increased DFS in addition to acceptable adverse events. This recommendation places a higher value on treating cancer in a single surgery with optimal margins and minimizing the risk of recurrence than potential additional surgery and the associated risk of adverse events that accompany a second surgery for margins. The benefits of BCS with negative margins are considered greater than the harms, and the evidence is generalizable to the entire target population. Positive and close margins after mastectomy are quite rare, and evidence was limited regarding the optimal treatment management in that circumstance.

In all cases of DCIS, the available evidence suggests that a margin width of 2 mm minimizes the risk of recurrence, and a wider margin width is not indicative of a lower risk of LR. Therefore, the Working Group made the recommendation in favor of a margin width of at least 2 mm to minimize the risk of LR. It remains entirely appropriate in pathology practice to report only DCIS at the inked margin as “positive” and to provide distance to the closest margin(s) when margins are negative.

#### 3.1.7. Recommendation 3

• In patients with negative margins (at least 2 mm) undergoing BCS routine, additional surgery may not be warranted if they are undergoing RT, but the re-excision of wider excisions should be considered if they forego RT.
• In patients with close margins (2 mm) from BCS or mastectomy, a discussion should occur with the patient to weigh the risks of further surgery (re-excision or mastectomy) with the risk of recurrence for the individual patient. Patients with close margins where re-excision versus boost RT is being considered should be discussed in multidisciplinary discussions involving surgical and radiation oncologists to tailor the optimal treatment plan.
• In patients with positive margins from BCS or mastectomy, re-excision should be considered as soon as information is available.

#### 3.1.8. Qualifying Statements for Recommendation 3

• The potential risks of cancer recurrence versus additional surgical procedures should be discussed between the patient and surgeon.
• The benefits and harms of re-excision may vary depending on patient and disease characteristics such as patient factors/comorbidities, patient preferences, tumor characteristics, life expectancy, and any contraindication to or unwillingness to receive RT.
• For patients whose close or positive margins are anterior or posterior, there may be no benefit to re-excision in areas where there is no remaining breast tissue. Multidisciplinary discussion is encouraged to discuss the benefits of boosting RT.
• DCIS-M should be considered as DCIS when considering margin width and additional surgery.

#### 3.1.9. Key Evidence and Justification for Recommendation 3

There were no RCTs found comparing re-excision to no re-excision in patients with suboptimal margin (close or positive) after the initial surgery of BCS or mastectomy; therefore, there is no strong evidence for one treatment strategy over another. It was the consensus of the Working Group that further surgery may be warranted in order to minimize the risk of recurrence, which is highest in patients with positive margins, less so in close margins, and even less with negative margins [10,23,31]. While such a procedure can be both physically and mentally challenging for the patient, the benefits of such a treatment plan outweigh the risks. Many factors such as comorbidity, patient preferences, re-excision cosmetic impact, life expectancy, tumor characteristics, and any contraindication to or unwillingness to receive RT should be considered before proceeding with re-excision.

### 3.2. Primary Treatment of DCIS: Surgical Treatment and/or RT

#### 3.2.1. Recommendation 4

• There are insufficient data to recommend or not recommend molecular profile testing as routine standard practice in women with DCIS. Molecular profile testing should only be performed as part of a research study.

#### 3.2.2. Key Evidence and Justification for Recommendation 4

Two full-text publications reported on the outcome of recurrence on the molecular profile test Oncotype DX [22,28] and one abstract on DCISionRT [27]. The certainty of the evidence for each intervention comparison was considered “very low”, and the results are very highly variable due to the small number of LR events, limited follow-up time, and wide confidence intervals. In addition, one study included only patients with negative margins. It was the consensus of the Working Group that due to the lack of mature data, molecular profile testing could not be recommended or not recommended as a routine standard practice in women with DCIS. The Working Group recognizes that ongoing trials may provide further information in this area in the next several years (See Appendix A.4 for a list of ongoing trials).

#### 3.2.3. Recommendation 5

• Women with DCIS who have undergone BCS with negative margins should be offered adjuvant whole breast irradiation (WBI, regardless of the grade of DCIS).
• For women with DCIS who have undergone BCS with close margins (<2 mm) for whom re-excision surgery was not performed, multidisciplinary discussion regarding the option of radiation boost in addition to WBI to optimize local control should occur.
• Postmastectomy radiation therapy (PMRT) is not indicated for women with DCIS who have undergone mastectomy but may be considered if there are multiple positive margins (tumor on ink) that cannot be surgically excised.

#### 3.2.4. Qualifying Statements for Recommendation 5

• The potential risks of cancer recurrence versus adjuvant irradiation should be discussed between the patient and clinicians post BCS and postmastectomy. Fully informed patients with low-risk DCIS may prefer to avoid RT.
• Hypofractionated RT (HFRT) of 42.5 Gy in 16 fractions for 3.5 weeks or an equivalent regimen (e.g., 40 Gy in 15 fractions in 3 weeks) should be offered. We acknowledge that even shorter regimens (e.g., 26 Gy in 5 fractions in 1 week) may also be offered (see recommendation justification below).
• Although there was a benefit for boost across all patients’ subtypes, the dose of 16 Gy in eight fractions may be associated with increased toxicity over time, and the risks and benefits of a boost need to be weighed, as well as other potential options using lower doses (10 Gy/4 to 5 fractions to 16 Gy/8 fractions).
• The risk of adverse effects associated with tumor bed boosts following WBI should be discussed.
• For patients with low-risk DCIS, patients with mammographically detected low or intermediate-grade DCIS measuring 2 cm or less and who are 40 years old or older, partial breast irradiation (PBI) may be considered.
• It was the expert opinion of the Working Group that one could safely extrapolate the benefits of adjuvant radiation with more than 5 cm of DCIS where complete excision is achieved. Patients were originally excluded from these studies [14,18] because advanced surgical breast conserving techniques did not exist at that time (e.g., oncoplastic reduction mammoplasty). In these cases, multidisciplinary discussion is encouraged for those presenting with more than 5 cm of disease.
• There is a lack of data on adding adjuvant chest wall irradiation after mastectomy, as a close or positive margin after mastectomy is quite rare. There are, however, studies showing a higher risk of LR in patients with close or positive margins compared to negative margins [9,29]. It is not clear if PMRT is beneficial in this setting, given there are few studies that specifically examine the LR risk postmastectomy with positive margins with or without PMRT. Furthermore, while close or positive margins increase the risk of LR in this setting, overall, the LR risk is relatively low (5.3%) [9]. It was the expert opinion that PMRT is not indicated in this setting, but it is reasonable to consider chest wall irradiation in patients who have undergone mastectomy with multiple positive margins (tumor on ink) that cannot be surgically excised.

#### 3.2.5. Key Evidence and Justification for Recommendation 5

One systematic review [12] and 12 full text publications on six RCT trials [14,15,16,17,18,19,21,25,26,32,33,34] included patients undergoing BCS, comparing either RT versus none, tumor boost versus none, conventional RT vs. HFRT, or partial breast irradiation (PBI) vs. WBI. There were no publications meeting our inclusion criteria for mastectomy patients.

These recommendations place a higher value on avoiding cancer recurrence than an increased risk of treatment-related adverse events. With the addition of RT, the desirable effects were moderate (i.e., significant differences in recurrence rates, but no significant differences in survival rates), and the undesirable effects were moderate (i.e., there were clinically meaningful differences in adverse events) in comparison to no RT. It was the consensus of the Working Group that the significant reduction in recurrence rates outweighed the adverse effects of adjuvant breast irradiation. The potential risks of cancer recurrence versus the potential adverse effects of breast irradiation should be discussed between the patient and surgeon.

Recently, the BIG 3-07/TROG 07.01 phase III RCT demonstrated that the addition of a boost dose to WBI resulted in a lower recurrence rate among non-low-risk DCIS patients undergoing BCS [28]. There is high certainty in the evidence to suggest it could reduce recurrence rates but at the additional risk of increased treatment-related adverse events such as grade 2 or higher breast pain and induration [15,16]. In the BIG 3-07/TROG 07.01 trial, a boost dose of 16 Gy in eight fractions was used [16]. There was a potentially greater benefit in adding boost RT for patients with larger tumor sizes and other risk factors such as high grade and younger age. There was some evidence to suggest no difference in survival with the additional tumor boost; however, there is no long-term evidence yet of survival data (only 5 years follow-up).

Evidence from the BIG 3-07/RTOG 07.01 suggests that moderately, HFRT is as effective as CRT in women with non-low-risk DCIS after BCS, where fewer, larger radiation doses over a shorter period were safe and as effective as CRT [16]. It was the consensus of the Working Group that an HFRT of 42.5 Gy in 16 fractions or an equivalent regimen (e.g., 40 Gy in 15 fractions in 3 weeks) should be offered to patients. The Working Group acknowledged that shorter regimens (e.g., 26 Gy in 5 fractions) might also be offered, such as those used in the FAST-Forward randomized trial for invasive breast cancer, which showed that 26 Gy in five fractions over one week was non-inferior to moderate HFRT both for local control and normal tissue toxicity at five years [39].

Moreover, the evidence suggests that PBI is as effective as WBI in terms of recurrence rates among patients with DCIS. A high-level systematic review with meta-analysis of the National Surgical Adjuvant Breast and Bowel Project (NSABP)-39 [40] and RAPID [41] studies did not observe a significant difference in 10-year recurrence rates among patients treated with PBI compared with WBI. There was no further information on which DCIS patients may or may not be favorable candidates for PBI. It was the expert opinion of the Working Group that adjuvant PBI after BCS may be considered in carefully selected patients with low-risk DCIS meeting all aspects, as defined by the RTOG 9804 criteria of mammographically detected low or intermediate-grade DCIS, measuring less than 2.5 cm with margins greater or equal to 3 mm. This is consistent with the ASTRO guideline [42,43]. There were two other RCTs on PBI after BCS for early-stage breast cancer that did not meet prespecified criteria of separating DCIS and invasive disease (University of Florence and GEC-ESTRO) that show that PBI has similar recurrence rates as WBI.

### 3.3. Management of DCIS After Primary Treatment

#### 3.3.1. Recommendation 6

• The risks and benefits of endocrine therapy, either tamoxifen or an aromatase inhibitor, after BCS should be discussed for women with estrogen receptor (ER)-positive DCIS.

#### 3.3.2. Qualifying Statements for Recommendation 6

• This does not pertain to women with bilateral mastectomy for DCIS but is relevant for unilateral mastectomy, whether they have had or not had RT.
• Possible risks could include increased toxicity and adverse events with no survival benefit. There are higher reported rates of endometrial, ovarian, and nonmelanoma skin cancer in tamoxifen use and higher rates of fractures, strokes, and transient ischemic events with aromatase inhibitor use.
• Possible benefits include the prevention of ipsilateral recurrences and contralateral events. This is true for in situ/preinvasive and invasive diseases.
• Tamoxifen, or an aromatase inhibitor for five years taken as a once-daily tablet, is the surgical standard of care in the adjuvant setting postsurgical resection.
• For postmenopausal women younger than 60 years, there may be a greater benefit to anastrozole compared to tamoxifen.
• Shared decision-making process to discuss the individual risk patient value, preference of agent, duration of agent, and cost.

#### 3.3.3. Key Evidence and Justification for Recommendation 6

Four full text publications and one abstract of patients undergoing BCS comparing tamoxifen versus none (or placebo) and tamoxifen versus anastrozole were included [13,17,20,24,30]. There were no trials meeting our inclusion criteria for mastectomy patients.

There was moderate certainty in the evidence to suggest a benefit with the addition of tamoxifen in reducing recurrence rates and contralateral events in women treated with BCS, particularly in women who are ER positive [13]. Results from the IBIS-II and NSABP-B-35 studies suggest no significant difference between tamoxifen or anastrozole as a choice of endocrine therapy in the management of DCIS to reduce recurrence rates. For postmenopausal women younger than 60 years, there may be a greater benefit to anastrozole compared to tamoxifen. While there are possible benefits in the prevention of recurrence events, there was high certainty in the evidence that there are also increased risks of toxicity and adverse events, such as endometrial cancer, deep vein thrombosis, and transient ischemic attack. It was the consensus of the Working Group that the risks and benefits of endocrine therapy, either tamoxifen or an aromatase inhibitor after BCS, should be discussed with ER-positive DCIS patients.

The Working Group acknowledges that a lower dose of tamoxifen for a shorter period and reduced dose (i.e., 5 mg daily tamoxifen for 3 years) may also be an option for reducing recurrence in the hormone-sensitive breast with similar or slightly lower toxicity than a full dose; however, this study [44] did not meet prespecified criteria for inclusion in this systematic review. Physician-based preference or shared decision-making process should be employed to discuss each individual personal risk, the patient’s values, the preference of the agent, the duration of an agent, and the potential cost involved.

## 4. Discussion

This comprehensive guideline on the management of DCIS provides valuable insights that are useful in clinical decision making for healthcare providers. The management of a patient with DCIS can depend on a variety of factors, including the extent of the disease in relation to the patient’s breast size, the presence of genetic mutations, any contraindications to RT, and the patient’s overall health and preference. The recommendations made above were based on the surgical treatment options and/or RT, along with the management of DCI after primary treatment.

When implementing the recommendations, patient and societal resource availability should be considered. In some healthcare settings or geographical locations, the availability of resources for certain treatments, such as breast reconstruction, breast irradiation, and genetic testing, may be limited. This guideline does not cover diagnosis or staging (i.e., the methods of diagnosis including mammography, magnetic resonance imaging biopsy, histopathological evaluation, or the staging/classification of DCIS), follow-up and surveillance, quality of life and survivorship, or patient education. The systematic review inclusion criteria were limited to RCTs where there were RCTs available. In the absence of any RCTs, the inclusion of retrospective studies was included to provide some additional information.

### 4.1. Limitations

DCIS remains an area of active research. Continued research into molecular profiling may help identify which DCIS cases are likely to progress to invasive breast cancer and which can be safely managed with less aggressive treatment or active surveillance. Also, studies aimed to optimize the use of RT, including the investigation of shorter treatment regimens or targeted RT techniques (e.g., stereotactic body RT to minimize adverse side effects while maintaining effectiveness), are ongoing.

### 4.2. Review and Update

All documents are maintained and updated through an annual assessment and subsequent review process, and where appropriate, the addition of new literature to the original evidence. Further details can be found in the PEBC Document Assessment and Review Protocol. For the full 1–10 Version 2 guideline, systematic review, and subsequent updates, please visit the OH (CCO) website at https://www.cancercareontario.ca/en/guidelines-advice/types-of-cancer/breast (accessed on 21 July 2024).

## Appendix A

The appendix contains supplementary materials such as affiliations and conflict of interest declarations, responses to the Targeted Peer Review Questionnaire, Responses to the Professional Consultation Survey, and ongoing trials.

### Appendix A.2. Conflict of Interest of Internal Reviewers and Targeted Peer Reviewers

**EXPERT PANEL**		
**Name**	**Specialty**	**Declarations of Interest**
Anita Bane	Breast pathologist, Toronto General Hospital: University Health Network	None declared
Ryan Carlson	Radiation oncologist, Health Sciences North, Regional Cancer Program	None declared
Jessica Conway	Medical oncologist, Royal Victoria Hospital	None declared
Harriet Feilotter	Senior scientist- molecular pathology. Queen’s Cancer Research Institute	None declared
Samantha Fienberg	Radiologist. Cancer Care Ontario, Ontario Health	None declared
Leta Forbes	Medical oncologist, Cancer Care Ontario, Ontario Health	None declared
Sonal Gandhi	Medical oncologist, Odette Cancer Centre Sunnybrook Health Sciences Centre	Has received $500 or more in a single year in a consulting capacity for Lily AD BOARD, Agendia AD BOARD, AZ AD BOARD, and Novartis AD BOARD (stipends total ~$5000)
Renee Hanrahan	Medical oncologist, Collingwood General and Marine Hospital, Royal Victoria Regional Health Centre	None declared
Glykeria Martou	Assistant professor, Plastic Surgeon, Queen’s University, Kingston General Hospital Hotel Dieu Hospital	None declared
Francisco Perera	Radiation oncologist, London Regional Cancer Program	None declared
Christiaan Stevens	Radiation oncologist Royal Victoria Hospital	None declared
**REPORT APPROVAL PANEL**		
**Name**	**Specialty**	**Declarations of interest**
William K. Evans	Medical oncologist, Oncosynthesis Consulting Inc.	None declared
Michelle Ghert	Surgeon, Juravinski Cancer Centre, Hamilton Ontario, Canada	None declared
Jonanthan Sussman	Radiation oncologist, Juravinski Cancer Centre, Hamilton Ontario, Canada	None declared
**TARGETED PEER REVIEW**		
**Name**	**Specialty**	**Declarations of interest**
Petrina Causer	Radiologist, Community Hospital- North York General Hospital, Toronto, Ontario, Canada	None declared
Ralph George	Surgery. St. Michael’s Hospital, Toronto, Ontario, Canada	Has been a co-principal for the PET ABC study, which looked at PET for staging LABC.

### Appendix A.3. Key Results from the Targeted Peer Reviewer Questionnaire

	**Reviewer Ratings (N = 2)**				
**Question**	**Lowest Quality** **(1)**	**(2)**	**Neutral (3)**	**(4)**	**Highest Quality** **(5)**
1. Rate the guideline development methods.					2
2. Rate the guideline presentation.					2
3. Rate the guideline recommendations.					2
4. Rate the completeness of reporting.				1	1
5. Does this document provide sufficient information to inform your decisions? If not, what areas are missing?					2
6. Rate the overall quality of the guideline report.					2
	**Strongly Disagree** **(1)**	**(2)**	**Neutral (3)**	**(4)**	**Strongly Agree** **(5)**
7. I would make use of this guideline in my professional decisions.					2
8. I would recommend this guideline for use in practice.					2
9. What are the barriers or enablers to the implementation of this guideline report?	None listed.				
**Comments**	**Responses**				
1. The role of post operative imaging in the management of close and positive DCIS margins was not mentioned. Does more imaging to determine obvious residual disease play a role in the decision to re-excise vs. boost radiation?	We have added a qualifying statement to Recommendation 1: The use of imaging modalities to assess for residual disease in patients with positive markings post BCS is outside the scope of this guideline but the Working Group consensus favors positive margins being treated surgically given perceived low sensitivity for detecting residual disease versus postoperative changes in patients having undergone recent surgery with all imaging modalities.				

### Appendix A.4. Professional Consultation Feedback on the Draft Recommendations and Working Group Responses

**Comments**	**Responses**
1. The guideline did not discuss the emerging data on the role or HER2 Receptor status/treatment in DCIS	This study did not meet the prespecified criteria of this systematic review.
2. Suggest adding “evidence does not strong favor” to the beginning of Recommendation 1 and 6.	The Working Group has decided to leave the recommendations as is.
3. Suggest mentioning RT option after BCS for context in Recommendation 1 as an enabler to patients making the decision about BCS vs. mastectomy (and state “see Recommendation 5 below”)	We have added a phrase in a qualifying statement to indicate that RT options after BCS are covered in Recommendation 5 below.
4. Since there is a lack of conclusive evidence for Recommendation 4 consider adding “Molecular profile testing should be confined to ongoing research”	The Working Group has added this phrase to recommendation 4.
5. Would consider adding most recent trial on low dose tamoxifen (TAM-01)	This study did not meet the prespecified criteria of this systematic review as the number of DCIS patients comprised less than 80% of the patient population and did not provide a separate analysis. More information can be found on page 51.
6. Recommendation 5.1 should be reworded to give an age component with lower Grade DCIS as there is much discussion globally to de-escalate therapy for elderly women	The Working Group has decided to leave the recommendation as it. The potential risks and benefits of adjuvant irradiation should be discussed between individual patient and clinicians.

### Appendix A.5. Ongoing Trials (on 26 July 2023)

**Title and Protocol ID**	**Study Details and Status**
**Surgical treatment/Active Surveillance**	
A Randomized Phase 2 Study Comparing Surgical Excision Versus Neoadjuvant Radiotherapy Followed by a Delayed Surgical Excision of Ductal Carcinoma In Situ (NORDIS)-NCT03909282	Phase 2 trial surgical excision vs. neoadjuvant radiotherapy + a delayed surgical excision of DCIS (NORDIS; estimated time of completion 2025).
Impact of Neoadjuvant Hormonal Therapy on the Surgical Management of Extensive Ductal Carcinomas in Situ (NORNE001)- NCT04666961	Phase 2 trial investigating neoadjuvant tamoxifen or anastrozole and a delayed surgical excision of DCIS (estimated time of completion 2024).
Comparing an Operation to Monitoring, With or Without Endocrine Therapy (COMET) Trial For Low Risk DCIS--NCT02926911	Phase 3 prospective randomized trial comparing surgery +/− radiation with the choice of endocrine therapy and active monitoring with the choice of endocrine therapy (estimated time of completion 2028).
Management of Low-Risk (Grade I and II) DCIS (LORD)- NCT02492607	Nonrandomized trial examining wide local excision +RT or wide local excision or mastectomy vs. active surveillance (estimated time of completion 2029)
A trial comparing surgery with active monitoring in low risk DCIS (UK-LORIS)	A phase 3 trial comparing surgery (+/− RT and/or hormonal therapy) and active monitoring. Recruitment for this trial has ended (estimated time of completion unknown).
Prospective Evaluation of Breast-Conserving Surgery Alone in Low Risk Ductal Carcinoma in Situ (ELISA)-NCT04797299	Prospective cohort study to evaluate whether the combination of clinicopathological factors and the use of Oncotype DX DCIS score can avoid radiation in women with low-risk DCIS who have had BCS (estimated time of completion 2035).
Wide Excision Alone as Treatment for Ductal Carcinoma In Situ of the Breast-NCT00165256	Phase 2 study to determine if wide excision (surgical removal) alone is adequate treatment for small, grade 1 or 2, DCIS of the breast (estimated time of completion 2023) (surgery vs. observation)
**Management after DCIS after primary treatment**	
Radiotherapy Versus Low-Dose Tamoxifen Following Breast Conserving Surgery for Low Risk Breast Ductal Carcinoma in Situ -NCT04046159	Phase 3 trial comparing RT (50 Gy/25 fx or 40.05 Gy/15 fx) vs. low-dose tamoxifen (5 mg QD for 10 yrs) in low-risk and estrogen receptor-positive DCIS (estimated time of completion 2025)
Testing an Active Form of Tamoxifen (4-hydroxytamoxifen) Delivered through the Breast Skin to Control Ductal Carcinoma in Situ (DCIS) of the Breast-NCT02993159	Phase 2 trial comparing 2 mg once daily per breast of 4-hydroxytamoxifen topical gel vs. 20 mg daily oral tamoxifen citrate (estimated time of completion 2023)
Hypofractionated Partial Breast Irradiation in Treating Patients with Early Stage Breast Cancer-NCT03077841	Phase 2/3 trials comparing hypofractionated partial breast irradiation daily for 5 days (+possible 3 boost fractions at discretion of the doctor) vs. standard irradiation daily for 15 days (+possible 5 boost fractions at discretion of the doctor; estimated time of completion 2024).
Single-arm confirmatory trial of endocrine therapy alone for estrogen receptor-positive, low risk ductal carcinoma in situ of the breast (JCOG1505, LORETTA trial)	Trial comparing endocrine therapy alone vs. non in low-risk estrogen receptor positive patients (estimated time of completion unknown)
**Molecular Testing**	
The AUS-PREDICT Registry for DCIS Patients with DCISionRT Testing-NCT04916808	Prospective cohort study of patients diagnosed with DCIS and to create a database of patients, test results, treatment decisions, and outcomes to determine the utility of DCISionRT (estimated time of completion 2024)
The PREDICT Registry for DCIS Patients with DCISionRT Testing NCT03448926	Prospective cohort study of patients diagnosed with DCIS and to create a database of patients, test results, treatment decisions, and outcomes to determine the utility of DCISionRT (estimated time of completion 2025)
